# Integrative analysis of *in vivo* recording with single-cell RNA-seq data reveals molecular properties of light-sensitive neurons in mouse V1

**DOI:** 10.1007/s13238-020-00720-y

**Published:** 2020-04-29

**Authors:** Jianwei Liu, Mengdi Wang, Le Sun, Na Clara Pan, Changjiang Zhang, Junjing Zhang, Zhentao Zuo, Sheng He, Qian Wu, Xiaoqun Wang

**Affiliations:** 1 State Key Laboratory of Brain and Cognitive Science, CAS Center for Excellence in Brain Science and Intelligence Technology, Institute of Brain-Intelligence Technology (Shanghai), Institute of Biophysics, Chinese Academy of Sciences, Beijing, 100101 China; 2 State Key Laboratory of Cognitive Neuroscience and Learning, Beijing Normal University, Beijing, 100875 China; 3 University of Chinese Academy of Sciences, Beijing, 100049 China; 4 Institute for Stem Cell and Regeneration, Chinese Academy of Sciences, Beijing, 100101 China; 5 IDG/McGovern Institute for Brain Research, Beijing Normal University, Beijing, 100875 China; 6 Advanced Innovation Center for Human Brain Protection, Beijing Institute for Brain Disorders, Capital Medical University, Beijing, 100069 China

**Keywords:** light sensitivity, *vivo*-seq, patch-seq, calcium imaging *in vivo*, whole cell recording *in vivo*

## Abstract

Vision formation is classically based on projections from retinal ganglion cells (RGC) to the lateral geniculate nucleus (LGN) and the primary visual cortex (V1). Neurons in the mouse V1 are tuned to light stimuli. Although the cellular information of the retina and the LGN has been widely studied, the transcriptome profiles of single light-stimulated neuron in V1 remain unknown. In our study, in vivo calcium imaging and whole-cell electrophysiological patch-clamp recording were utilized to identify 53 individual cells from layer 2/3 of V1 as light-sensitive (LS) or non-light-sensitive (NS) by single-cell light-evoked calcium evaluation and action potential spiking. The contents of each cell after functional tests were aspirated *in vivo* through a patch-clamp pipette for mRNA sequencing. Moreover, the three-dimensional (3-D) morphological characterizations of the neurons were reconstructed in a live mouse after the whole-cell recordings. Our sequencing results indicated that V1 neurons with a high expression of genes related to transmission regulation, such as *Rtn4r* and *Rgs7,* and genes involved in membrane transport, such as Na^+^/K^+^ ATPase and NMDA-type glutamatergic receptors, preferentially responded to light stimulation. Furthermore, an antagonist that blocks *Rtn4r* signals could inactivate the neuronal responses to light stimulation in live mice. In conclusion, our findings of the *vivo*-seq analysis indicate the key role of the strength of synaptic transmission possesses neurons in V1 of light sensory.

**Electronic supplementary material:**

The online version of this article (10.1007/s13238-020-00720-y) contains supplementary material, which is available to authorized users.

## Introduction

Visual perception involves the activity of neurons in the cerebral cortex. The ‘retino-geniculo-cortical’ pathway indicates the best-known route for visual information (Chalupa, 2003). In the rodent primary visual cortex (V1), pyramidal cells together with inhibitory neurons confer highly specific visual features, such as light sensitivity and perceptual discrimination (Niell and Stryker 2008; Bock et al., 2011). It has been found that pyramidal cells in layer 2/3 with a similar light orientation preferentially form synapses with each other (Lee et al., 2016). Specific types of interneurons in V1, such as parvalbumin (PV)- and somatostatin (SST)-positive interneurons, are also essential for modifying neuronal feature selectivity and improving perceptual discrimination (Lee et al., 2012; Hagihara and Ohki 2013). Recently, studies have indicated that direction-selective retinal ganglion cells (RGCs) deliver direction-tuned and orientation-tuned signals to superficial V1 (Cruz-Martin et al., 2014). However, the molecular properties of the light-sensitive neurons in the V1 cortex remain unclear. Single cell RNA sequencing (scRNA-seq) techniques give the potential to reveal the genetic characteristics of single neuron (Liu et al., 2017; Fan et al., 2018; Zhong et al., 2018). Especially, high-throughput single-cell RNA sequencing has been applied to identify significant and divergent transcriptional responses of V1 neurons to sensory experience (Hrvatin et al., 2018).

The patch-seq technique, a method that combines whole-cell electrophysiological patch clamp recording and single-cell RNA sequencing, was presented to link the molecular profile to its corresponding electrophysiological and morphological counterparts in individual neurons (Cadwell et al., 2016). To date, the patch-seq technique has been widely used on mouse brain slices (Fuzik et al., 2016) or single neurons in culture (Bardy et al., 2016; Chen et al., 2016; Li et al., 2016). However, the physical environment during whole-cell recording on brain slices is substantially different from the *in vivo* environment and the local circuitry related to acute stimulation is not able to be tested in the brain slices.

To address these questions, we developed a method for functional *in vivo* single cell RNA-seq (*vivo*-seq) analysis for combining intracellular calcium imaging, *in vivo* whole-cell patch clamp recording, and high-quality RNA sequencing of individual neurons at layer 2/3 of the mouse V1 cortex while the mouse was stimulated via light grating under light anesthetization. By labeling the cells at layer 2/3 of the mouse V1 via calcium indicator, the intracellular calcium response and action potential firing were recorded synchronously to the light stimuli. After the identification of the transient light response neurons, the target neurons were attracted and further mRNA sequenced. The *vivo*-seq analysis identified the molecular biomarkers that were involved in the signaling pathways of sensing light in V1, and suggested that the transmission strength and plasticity of synapse in V1 played an important role in light sensitivity of V1 neurons.

## Results

### *In vivo* recording identified neurons in layer 2/3 of V1 could be identified as LS- and NS-neurons

**Figure 1:**
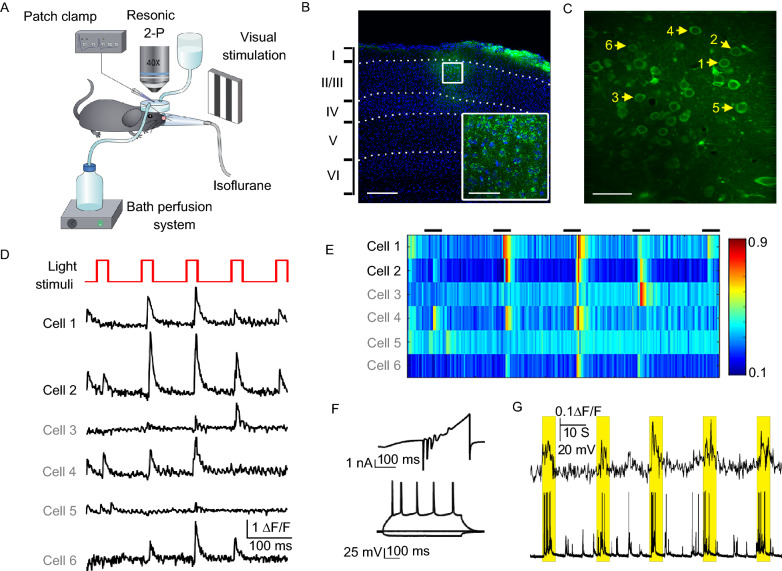
***In vivo*recording of light-sensitive neurons in layer 2/3 of V1**. (A) Overall view of the experimental arrangement of *in vivo* physiological recording with screening-evoked light stimulation. (B) Cal-520 AM labeled neurons in layer 2/3 of V1. Green, Cal-520 staining; blue, DAPI; white dotted line, laminar delimitation. Scale bar, 200 μm; scale bar of the insert, 50 μm. (C) Calcium imaging of Cal-520 AM labeling, with yellow arrows indicating the six target neurons. Scale bar, 50 μm. (D) Calcium response to light stimuli of six neurons in (C). Top panel (red): visual stimulation sequence starts with a stationary period of square-wave gating for 5 s, with an inter-pulse interval of 15 s. Cells 1 and 2 (black label) were defined as light-sensitive, and cells 3–6 (grey label) were excluded according to our inclusion criteria described in the methods. (E) Heat map of the calcium responses to light stimulation of the six neurons in (C). (F) Electrophysiological recording of a light-sensitive neuron. Upper panel: whole-cell current evoked by a 450 ms ramp voltage from −120 mV to +80 mV. Bottom panel: patterned action potential evoked by a stepped 500-ms current injection (−80 pA, 0 pA and 220 pA). (G) Representative dual recording of response (upper panel) and action potential firing (bottom panel) of one light-sensitive neuron. The yellow rectangle indicates the light stimulation period

In our *vivo*-seq system (Fig. 1A), the intracellular calcium activity from individual neurons at layer 2/3 of the mouse V1 cortex were recorded while the mouse was stimulated via light grating under light anesthetization. The cells at layer 2/3 of the V1 cortex were labeled with the Ca^2+^ indicator Cal-520 AM (Figs. 1B and S1A), which shows sufficient sensitivity for the detection of a single action potential and a high signal-to-noise ratio (Li et al., 2017a, b). The sensory-induced calcium signal can be legibly detected in V1 cortical neurons of a lightly anesthetized mouse (Cruz-Martin et al., 2017). The right eye of the mouse was administered a 5-s visual stimulation of black-white drifting square-wave grating, with an inter-stimulation interval of 15 s and 5 repetitions of the visual stimulation in total. The calcium signals at a depth of ~120 μm in the V1 cortex were detected and recorded at a frequency of 30 frames/s, via a two-photon microscope equipped with two photomultiplier tubes (PMT) (Fig. 1C). The calcium response to the 5-time repeated visual stimulations was calculated immediately by the manual MATLAB (R2016a, MathWorks) program (Fig. 1D and 1E). We defined a positive calcium response as having a calcium amplitude above the threshold (SNR = 2) at the moment of light stimulation. A cell that showed ≥4 positive calcium responses in one round of testing with 5 repetitions of light stimulation was classified as a light-sensitive (LS) cell (Cell 1 and Cell 2). Non-light-sensitive (NS) cells were cells with ≤1 positive response to the light stimulation. The identified cells were further recorded and imaged, and the soma was extracted for RNA sequencing (Supplementary Video S1).

To verify the calcium response evoked by the transient light stimuli, electrophysiological whole cell recording *in vivo* was applied. We used the ‘red’ channel for Texas Red fluorescence (620/60 nm) to trace the electrode filled with Texas Red and confirm the located neuron by its shadow. As the patch-clamp pipette (with a resistance of 7–10 MΩ) was approaching a target cell, we continuously ejected a solution that contained Texas Red and Neurobiotin from the tip with positive air pressure. When the electrode tip was sufficiently close to the target cell, we stopped ejection of the dye and applied negative air pressure to establish tight membrane sealing (> 1 GΩ), which was sustained for at least 2 min. A subsequent negative pressure was applied to rupture the membrane to form the whole-cell configuration, with an indication that the Texas Red diffused into the cytoplasm (Supplementary Video S2). A ramped I-V curve was applied to test the whole-cell current, including the inward sodium/calcium current and the outward potassium current (Fig. 1F, upper panel). The action potential firing was evoked by a stepped current injection (Fig. 1F, bottom panel). Furthermore, the calcium spikes and action potential were recorded at the same time as the light stimulation *in vivo*, showing the synchronic electrophysiological responses of a V1 cortical cell in response to light (Fig. 1G and Supplementary Video S3).

### Electrophysiological and morphological analysis of LS- and NS-neurons

**Figure 2:**
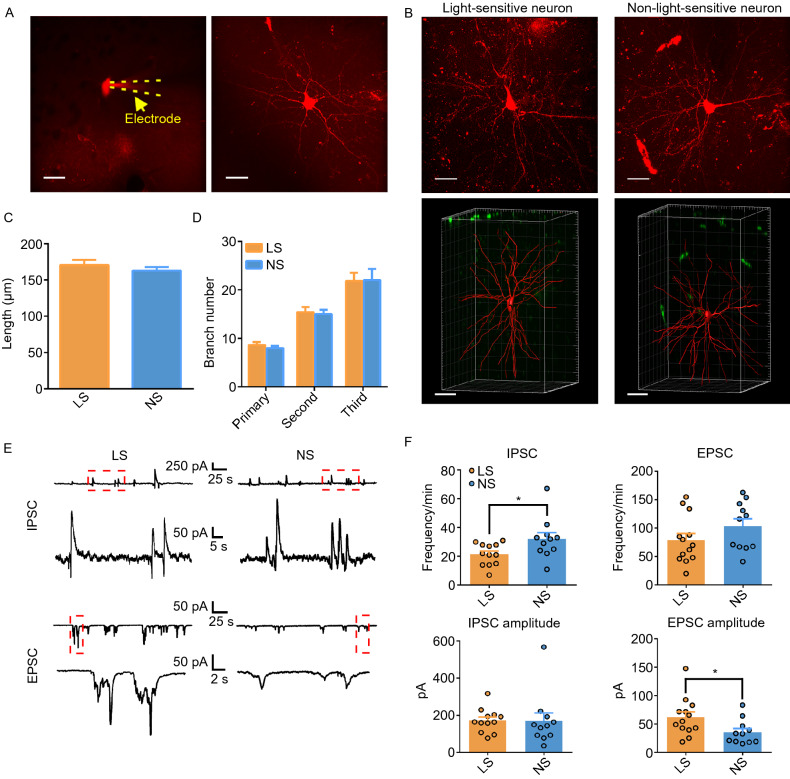
**Morphological analysis of LS- and NS-neurons in layer 2/3 of V1**. (A) Morphology of a patch clamp recorded neuron using resonic two-photon imaging. Left: Texas Red perfused into the cytoplasm through the electrode (yellow dotted line). Scale bar, 20 μm. (B) Morphology of the recorded neurons of the LS- and NS-groups under a two-photon microscope. Upper panel, Texas Red under *in vivo* imaging, scale bar: 20 µm; bottom panel, 3-D reconstruction of respective neurons (in red) with the background (in green). Scale bar, 30 μm. (C and D) Statistics of dendritic arbors of LS- and NS- neurons in V1. The dendritic morphology was assessed by quantifying (C) the distance from the soma to the longest dendritic terminal (*n* = 8 for LS and *n* = 7 for NS) and (D) the maximum number of primary, secondary and third intersections (*n* = 5 for LS and *n* = 5 for NS). Error bar, mean ± s.e.m. The significance was estimated based on the corrected *P* value using the unpaired *t*-test, *P* > 0.05 for non-significance. (E) Representative trace of spontaneous IPSCs (upper) and EPSCs (bottom) from light-sensitive neuron and non-light-sensitive neuron, respectively. (F) Statistics of IPSC frequency, IPSC amplitude (*n* = 12 for LS and *n* = 11 for NS), EPSC frequency and IPSC amplitude (*n* = 13 for LS and *n* = 11 for NS). Error bar, mean ± s.e.m. The significance was estimated based on the corrected *P* value using the unpaired *t*-test, *P* > 0.05 for non-significance

Under the whole-cell patch clamp configuration, the Texas Red diffused into the cytoplasm (Fig. 2A, left panels). The morphology of the NS- and LS-neurons could be immediately imaged under the ‘red’ channel for Texas Red fluorescence in the two-photon microscope (Fig. 2B). The three-dimensional cellular morphology and structure of neurons were reconstructed using the z stack images and Imaris (Fig. 2A, right panel and Supplementary Video S4). In addition, the location of the neuron in the V1 was confirmed by the Neurobiotin staining after the *in vivo* recording (Fig. S1B), and the arbor complexity was quantified by the stratus area and dendritic complexity after the three-dimensional reconstitution. The distance from the soma to the longest dendritic terminal was calculated as the stratus area, and the dendritic complexity was determined by the numbers of primary, secondary and third dendritic intersections. In conclusion, there were no significant differences in the area under the longest dendrite and the dendritic complexity between NS- and LS-neurons (Fig. 2C and 2D). Next, we compared the electrophysiological properties of LS- and NS-neurons. The statistical analyses of capacitance and input resistance of the recorded neurons showed no difference (Fig. S2). Next, we recoded IPSC at 0 mV of holding potential and EPSC at −70 mV of holding potential form the two groups of neurons, respectively (Fig. 2E). We found that frequency of IPSC was higher but the amplitude of EPSC was lower in NS cells than LS cells (Fig. 2F).

### Molecular properties of LS cells in V1

**Figure 3:**
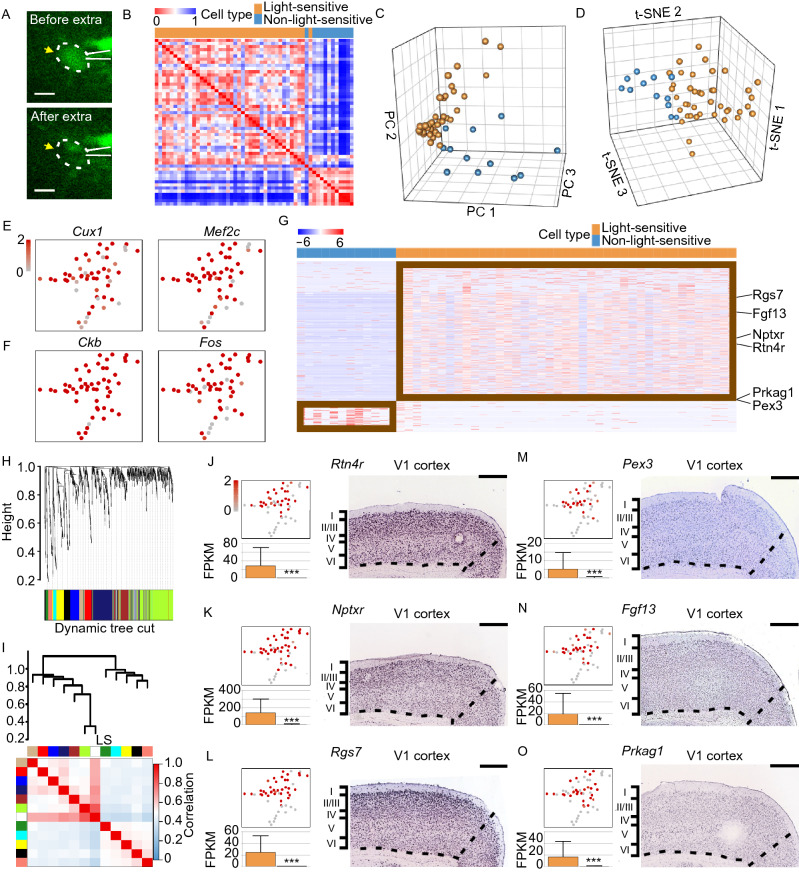
**Single-neuron transcriptome profile**. (A) Example of a neuron before and after soma extraction. The dotted line indicates the location of the soma, and the electrode is shown as white lines. Scale bar, 20 μm. (B) Pearson correlation heatmap of 53 cells from V1 of 24 mice. Clustering analysis separates light-sensitive (LS) and non-light-sensitive (NS) neurons. (C) Three-dimensional principal component analysis (PCA) of expression profiles of 20,183 genes detected in 53 sampled cells. (D) Three-dimensional representation of 53 samples using t-SNE. Cells are colored according to clustering as LS- and NS-cells. (E and F) Marker gene expression by cortical layer 2/3 neurons (E) and in primary visual cortical cells (F) overlaid onto the two-dimensional t-SNE. (G) Heatmap of log2 transformed gene expression of differentially expressed genes. Genes with P_adj_ < 0.05 and log2FoldChange > 2.5 or log2FoldChange < −2.5 were identified as differentially expressed genes. The differentially expressed genes were ranked in descending order based on their log2FoldChange value. (H) Gene dendrogram obtained by average linkage hierarchical clustering. The color underneath the row corresponds to the module assignment; each color represents a different assigned module. (I) Hierarchical clustering dendrogram (upper panel) and correlation heatmap (bottom panel) of the module eigengenes and the sample traits. Highly positive correlated branches were grouped together. The light-sensitive event (labeled as the white module) was correlated with colored gene modules in (H). Each cell in the matrix contains the corresponding correlation and *P*-value. Blue color represents a negative correlation, while red color represents a positive correlation. (J–O) Identification of differentially expressed genes between LS- and NS-neurons. Gene expression was overlaid onto the two-dimensional t-SNE, colored according to the FPKM normalized gene expression level. Expression quantitation is represented on the histogram as the mean ± s.d. The significance was estimated based on the *P* value computed by Deseq2, ** P_adj_ < 0.01, *** P_adj_<0.001. The expression pattern in V1 is shown as an ISH image (from Allen Brain Atlas). Scale bar, 420 μm

Since the morphological properties of LS- and NS-neurons have no significance but electrophysiological properties do, the transcriptomic profile of the two groups neurons would be further considered. For single-cell RNA sequencing, an autoclaved RNase-free pipette (with a resistance of 2–3 MΩ) was made to approach a target cell after calcium spike recording as previously described. When the electrode tip was sufficiently close to the target cell, we quickly sucked the soma into the pipette and collected the sample immediately after confirming that the soma entered by breaking the pipette tip (Fig. 3A and Supplementary Video S1). The internal pipette solution that contained the RNA-free pipette solution had an optimal volume of less than 1 μL (Cadwell et al., 2016).

After harvesting the cell contents, the single-cell mRNA was converted to cDNA and used to generate sequencing libraries following the protocol of Smart-seq2 (Cadwell et al., 2016). Cells with a low cDNA concentration (<1,000 pg/μL) (Fig. S3A) and poor sequencing quality (<1,000 genes) (Fig. S3B) were excluded from further analysis (3/63 cells). Cells with a high mitochondrial RNA fraction (> 25%) were also omitted (2/60 cells) (Fig. S3C). Moreover, one cell, which coexpressed a series of markers of different cell types, was considered as a contaminated cell. Hence, each cells was given co-expression scores and cells with scores greater than zero were discarded as well (5/58) (Fig. S3D).

In total, 83 neurons were selected from 89 recording cells of 24 mice. After implementing the previously described calcium response criteria, 63 cells remained. For scRNA sequencing, 53 of 63 cells (84.1%) passed the quality control, including 41 light-sensitive (LS) cells and 12 non-light-sensitive (NS) cells. We detected 5,432 and 4,111 genes, on average, for which the fragments per kilobase of transcript per million total reads (FPKM) was greater than 1 or 5 per cell, respectively (Fig. S3E), with a median Pearson correlation of 0.58 for LS cells and 0.43 for NS cells (Fig. S3F), which indicates that NS may be more diverse than LS cells.

Of 53 screened cells, two groups of cells were demonstrated by Pearson-correlation-based classification, which were mostly consistent with our functional definition of the LS- and NS-cells (Fig. 3B), suggesting that cells with the same functions may have similar transcriptomic profiles. After reducing the dimension of the expression profile of 20,183 genes detected in 53 cells, the LS- and NS-cells were clustered into two groups, using principal component analyses (PCA) (Fig. 3C) or t-distributed stochastic neighborhood embedding (t-SNE) algorithm (Fig. 3D). This clustering was not due to cDNA concentration deviation or experimental batch effects (Fig. S3G and S3H). Transcriptome analysis indicated that all cells were confirmed as neurons because they expressed marker genes of pyramidal cells (*Neurod1*, *Neurod2*, and *Emx1*) or interneurons (*Calb1*, *Cck*, and *Slc6a1*) (Fig. S4A). These neurons demonstrated high expression levels of the genes *Cux1* (cut like homeobox 1), *Cux2* (cut like homeobox 2), and *Mef2c* (myocyte-specific enhancer-binding factor 2) (Figs. 3E and S4B), which indicates that these are cells located in cortical layer 2/3 of adult mice (Leifer et al., 1993; Moroni et al., 2009; Juliandi et al., 2012). Moreover, these cells also exhibited high expressions of *Ckb* (Creatine kinase B), *Fos* (c-Fos) and *Junb* (Jun-B) (Figs. 3F and S4C), which were reported in mouse V1 in a recent study using high-throughput scRNA-seq (Hrvatin et al., 2018).

To identify the molecular properties involved in the light sensitivity of neurons, the differentially expressed genes (DEGs) between the LS neurons and NS neurons were analyzed. Genes with P_adj_ < 0.05 and log2FoldChange > 2.5 or log2FoldChange < −2.5 were identified as DEGs (Fig. 3G). WGCNA was used to divide the DEGs into 11 modules by linkage hierarchical clustering, and each module showed independent validation to each other (Fig. 3H). To further quantify the entire modules, we consider the LS- and NS-events as a new module (white module), thus adding up to 12 modules via digitalizing. We subsequently calculate their eigengenes and cluster them onto their correlation (Fig. 3I). The twelve modules yield two main clusters: one cluster contained five modules that were uncorrelated to the LS events, while the other cluster contained six modules that included the LS event module. Notably, the genes in the light green module are highly positively correlated with light-sensitive events in the module-trait association diagram (correlation coefficient = 0.65). *Rtn4r* (also referred to as *Ngr1*, the nogo receptor 1), *Nptxr* (the neuronal pentraxin receptor) and *Rgs7* were in the light green module, which indicates these genes are potentially responsible for light stimulation. Further analysis suggests that the mRNA expressions of these genes are high in LS cells compared to NS neurons and are expressed in V1 cortical neurons (Fig. 3J–L). *Rtn4r*^*+*^ cells were more in layer II/III than in deep layers, while *Nptxr*^*+*^ and *Rgs7*^*+*^ cells distributed relatively general in all layers. Importantly, these genes have been reported to mediate intralaminar synaptic plasticity in the visual pathway of light experience (Sia et al., 2007; Stephany et al., 2014; Gerber et al., 2016; Sarria et al., 2016; Stephany et al. 2016a, b; Lee et al., 2017), which indicates that synapses of light-active neurons in mouse V1 are potentially strengthened and maintained through competitive processes that require specific transcriptional regulation.

In addition to the well-studied genes involved in the visual system, we found that *Pex3* (peroxisomal biogenesis factor 3), *Fgf13* (fibroblast growth factor 13) and *Prkag1* (protein kinase AMP-activated non-catalytic subunit gamma 1) were also highly expressed in LS cells, which indicates their potential roles in regulating the light sensitivity of neurons in V1 (Fig. 3M−O). These genes contribute to the transmitter vesicle assembling (Lam et al., 2010), are responsible for axonal terminal growth (Wu et al., 2012), or manipulate the AMPK signal pathway (Gao et al., 1996), thus suggesting that LS neurons might be more compatible for light-evoked input transduction and signal processing in V1.

To further characterize the the LS- and NS-populations, we mapped *vivo*-seq cells with high-throughput scRNA-seq dataset of mouse visual cortex (Hrvatin et al., Hrvatin et al. 2018). Since our study focused on the neurons in layer 2/3 of mouse V1, we first extracted layer 2/3 excitatory neurons and inhibitory neurons from all cells based on the expression of known layer markers *Cux1*, *Cux2* and *Rorb* (Figs. S5A−D). Namely, neurons from clusters with high expression of *Cux1* and *Cux2* but rare expression of layer 4 marker gene *Rorb* were considered as layer 2/3 neurons (Figs. S5A−D) (Cubelos et al., 2015; Gray and Yao 2017). We conducted canonical correlation analysis (CCA) and reduced the dimensionality of both datasets onto the same two-dimensional space using t-SNE, which allowed the identification of 10 excitatory neuron and 4 interneuron clusters based on the expression of variable genes shared between both datasets (Figs. S5E and S5F). Namely, 78% LS neurons (32/41) and 75% NS neurons (9/12) were founded in the excitatory neuron clusters, while 22% LS neurons (9/41) and 25% NS neurons (3/12) were in the interneuron clusters (Fig. S5G). We then asked whether the LS- and NS- neurons preferentially fall into excitatory or interneuron subclusters. Thus, we counted the number of cells from vivo-seq in each subcluster and found that LS- and NS- neurons were mapped into 8 excitatory neuron subclusters and 3 interneuron subclusters but not the Ex-2, Ex-10 and Int-3 (Fig. S5H).

### *Rtn4r* was identified as the marker gene highly correlating to the light stimuli

**Figure 4:**
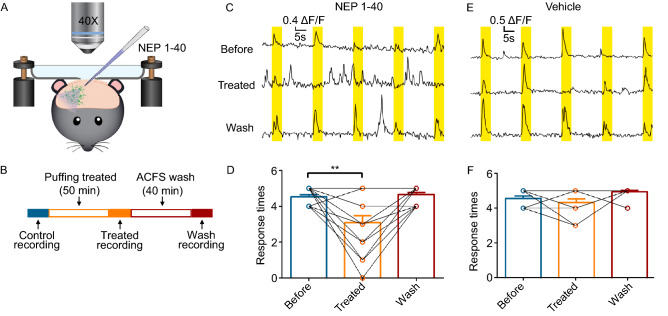
**Rtn4r regulates the light sensitivity of neurons in V1**. (A) Schematic of the *in vivo* puffing of NEP1-40. (B) Flow diagram of calcium recording with the NEP1-40/vehicle treatment. (C) Representative calcium response trace of LS neuron to light stimulation with the NEP1-40 treatment. The yellow rectangle indicates the light stimulation period. (D) Statistical histogram of the light response counts under the NEP1-40 application, *n* = 21 neurons from 5 mice. (E) Representative calcium response trace of LS neurons to light stimulation with the vehicle as a control, and the statistical histogram is shown in (F), *n* = 13 neurons from 3 mice. Error bar, mean ± s.e.m. The significance was estimated based on the corrected *P* value using the paired *t*-test, **P < 0.01

Among the up-regulated genes in LS neurons rather than NS neurons, *Rtn4r* (Nogo receptor 1, Ngr1) has been reported to mediate the intralaminar synaptic connectivity of visual cortical interneurons or a subclass of PV^+^ interneurons (Stephany et al., 2014; Stephany et al. 2016a, b). To verify whether Rtn4r disturbs the visual plasticity, an antagonist of Rtn4r, NEP1-40, was utilized (Fig. 4A and 4B). After the neuron was confirmed as a LS neuron, NEP1-40 was applied through the capillary puffing around the neuron constantly for 50 min. The light-evoked calcium responses were abolished after NEP1-40 treatment in these neurons, and the deficiency could be rescued after the drug was washed out in the same cells (Fig. 4C−F). The vehicle application, used as a control, could not change the light response of the LS neurons to the stimulation. Moreover, we also analyzed the cells, which responded to light stimulus less than twice (out of five times). Spontaneous calcium event counts were counted without light stimulation, before and after NEP1-40 or vehicle treatment, in these cells **(**Fig. S6). The results showed that the calcium activities were not blocked by either NEP1-40 (*n* = 15) or vehicle (*n* = 16) treatment, indicating that the neural basic calcium activities were not blocked by NEP1-40 (Fig. S6**)**. These results indicate that Rtn4r plays a key role in regulating the light sensing of LS neurons.

### Signals and neurotransmitters in LS neurons

**Figure 5:**
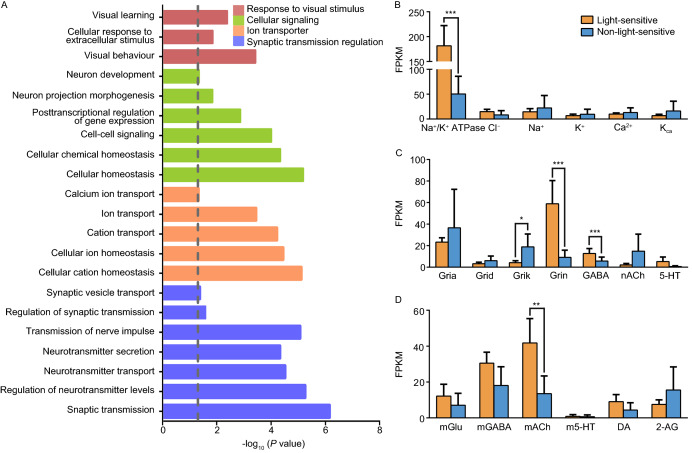
**Enriched gene ontology analysis**. (A) Histogram of the enriched GO terms of differentially expressed genes from the pairwise comparison of light-sensitive and non-light-sensitive cells. Dotted line, *P* = 0.05. (B–D) Quantitative expression of ion channel and membrane receptor genes in light-sensitive and non-light-sensitive cells. Genes include Na^+^/K^+^-ATPase, voltage-gated Cl^−^, Na^+^, K^+^, Ca^2+^, and Ca^2+^-activated K^+^ ion channels in (B); ligand-gated ionotropic channels of AMPA-, delta-, kainite- and NMDA-type glutamatergic receptors (Gria, Grid, Grik, and Grin, respectively); GABAergic (GABA), nicotinic acetylcholinergic (nACh) and serotonergic (5-HT) receptors in (C); metabolic glutamatergic (mGlu), GABAergic (mGABA) receptors, 5-HT, muscarinic acetylcholine (mACh), dopamine (DA) and 2-arachidonoylglycerol (2-AG) receptors in (D). Error bar, mean ± s.e.m. The significance was estimated based on the corrected P value using the unpaired t-test, *P < 0.05, **P < 0.01, ***P < 0.001

To further investigate the specific biological properties of LS neurons, gene ontology (GO) analysis was applied to the 200 most highly expressed genes of these cells. Genes were enriched into 21 GO terms and divided into 4 clusters, including response to the visual stimulus, cellular signaling, ion transporters and synaptic transmission regulation (Fig. 5A). Neurons dynamically tune their excitability via the integration of transmembrane ion channels and transporters and accomplish afferent and efferent communications by receptor-dependent transmitter release (Hucho 1993; Hille 2001; Arganda et al., 2007). Most of the information regarding the transcriptome profiles of transmembrane ion channels, ligand-gated inotropic channels and metabotropic (G protein-coupled) receptors of neurons was collected in our vivo-seq (Fig. 5B−D). We used the unpaired Welch’s two-sample t-test to determine whether two groups were significantly different from each other. Among the transmembrane proteins, the components that generate an action potential, including potassium, sodium and calcium channels, are consistent in the LS- and NS-groups.

Na^+^/K^+^-ATPase, which also functions as a signal transducer to regulate intracellular calcium in the retina (Plossl et al., 2017) and visual cortex (Meisami and Timiras 1974), showed higher mRNA expression in LS neurons than in NS neurons (*P* < 0.00001) (Fig. 5B). Considering that Na^+^/K^+^-ATPase participates in intracellular calcium signaling (Aperia et al., 2016), our data suggest that light stimulation triggers more calcium oscillations in LS neurons than in NS neurons. In addition, the subunits of the NMDA receptor (*P* < 0.00001) and kainite receptor (*P* < 0.05) are differentially expressed in the LS- and NS-groups. The ligands of both receptors are both glutamate, which can excite neurons. And Grin was highly expressed in LS neurons. The majority of ionotropic GABA receptors also have different expression levels in LS- and NS-neurons (*P* < 0.001) (Fig. 5C). We also observed that the NS neurons lack appreciable mRNA expression of the muscarinic acetylcholine receptors (mAChR), which have a significant expression in the LS neurons (*P* < 0.01) (Fig. 5D). Together, our results suggested that the sensory-evoked neurons possess stronger transmembrane conduction, including ion transport, synaptic transmission, and intracellular calcium variation.

## Discussion

In this work, we have illustrated that light-sensitive V1 neurons are functionally and transcriptionally correlated, and we also identified several molecular markers of these cells. Similar to the high-throughput RNA sequencing data sets in mouse V1 after chronic light exposure (Hrvatin et al., 2018), our transcriptome data showed that the sensory-stimulation-induced expression across most neurons contained regulator genes. The fastest known transcriptional response in any setting is the induction of immediate-early genes (IEGs), including *Fos*, *Junb* (Okuno, 2011). Not surprisingly, in our data, these neuronal activity response genes showed significantly higher expression in LS- than NS-neurons. In the brain, IEGs are linked to neural activity. These genes are constitutively expressed, but in weak basal levels (Kaczmarek and Chaudhuri 1997). Behavioral stimulation causes related neuron activity, which leads to DNA double-strand breaks in the promoter. As a result, neuronal IEGs are transcribed and the expression is dramatically increase (Flavell and Greenberg, 2008; Madabhushi et al., 2015; Hrvatin et al., 2018). Furthermore, genes that influence the transmission strength and synapse plasticity during the individual selection of superior neurons and elimination of inferior neurons such as *Rgs7*, also showed differential expression between the LS- and NS- groups (Gerber et al., 2016; Sarria et al., 2016). It is reported that the levels of RGS proteins determine key parameters of ON-bipolar cell light responses: kinetics, amplitude, and light sensitivity (Sarria et al., 2016). Another LS neuron specific gene, *Nptxr*, has been hypothesized to be involved in activity-dependent synaptic plasticity (Sia et al., 2007; Lee et al., 2017), as well as synaptic maturation through PV^+^ interneurons in V1 (Pelkey et al., 2015). Nptx1/2-expressing RGCs project to dorsal lateral geniculate nucleus (LGN) neurons (Bjartmar et al., 2006) and precisely map the visual cortex pattern (Cang et al., 2005). Thus, the high expression of *Nptxr* would indicate that it plays a key role in the visual ‘retino-geniculo-cortical’ pathway of light experience.

Rtn4r is attached to the plasma membrane by a lipid anchor. Once Rtn4r is activated by protein binding, it is proposed to transduce a signal from these ligands through one or more transmembrane “co-receptors”, such as Lingo, TROY (aliases: Tnfrsf19) and p75 (aliases: Ngfr), to activate the small GTPase RhoA (Stephany et al. 2016a, b). RhoA is known to play a key role in regulating neuronal morphology during development, synapse formation, spine dynamics and synaptic plasticity (Thomas et al.,^2018^). In our *vivo*-seq data, *RhoA* was also identified as a DEG that highly expressed in LS cells and clustered into the dark blue module in the gene dendrogram, which indicated close association with LS neurons. Thus, it is suggested that Rtn4r may play a critical role in regulating synaptic stability and elimination. However, how this Rtn4r-RhoA signaling pathway regulates anatomical plasticity and/or synaptic plasticity requires further exploration.

To follow the success of the Patch-seq by Fuzik et al and Cadwell et al (Cadwell et al., 2016; Fuzik et al., 2016), we further tried to connect the single cell transcriptome profiles with the sensory-evoked function *in vivo*. Our whole-cell recording in LS neurons indicated that the action potential firing, followed by the intracellular calcium evaluation, was strongly associated with the light stimulation on the eye. Our analysis data suggest the rapid physiological responses of LS neurons might be attributed to the higher expression level of transmembrane proteins, such as Na^+^/K^+^-ATPase, ionotropic glutamate and GABA subtype receptors. It is well known that glutamatergic transmission mainly relies on the N-methyl-D-aspartate (NMDA) receptor, α-amino-3-hydroxy-5-methyl-4-isoxazolepropionic acid (AMPA) receptors, delta receptors and kainite receptors expressed in neurons (Traynelis et al., 2010). Our single cell membrane channel analysis reveal that the expression of *Grin*, encode subunits of NMDA receptor (Paoletti et al., 2013), was higher in LS neurons (Fig. 5C). Considering the higher density of postsynaptic receptors can induce the higher amplitude of EPSC (O’Brien et al., 1998), the higher amplitude of EPSC may be caused by the higher expression level of *Grin* in LS neurons. Previous and current studies have indicated the mechanisms of NMDA receptor-induced LTD and LTP that account for key aspects of experience-dependent synaptic modification in the visual cortex (Kirkwood et al., 1996; Li et al., 2017a, b), as well as the GABAa receptor-dependent intracortical inhibitory inputs, are suggested to be involved in regulating the orientation tuning of the spike responses in the visual cortex (Pei et al., 1994; Ding et al., 2017). These results suggest that gene-transcription-regulated synaptic transmission dynamically promotes the light sensitivity of a subtype of V1 neurons. In addition, preliminary studies have reported that ACh application could increase neuronal orientation and direction selectivity in the cat V1 (Sillito and Kemp 1983; Sato et al., 1987); moreover, recent studies have also indicated that the mAChR could regulate the neuronal activity in V1 to improve the light contrast sensitivity in monkeys (Herrero et al., 2017), which was consistent with our sequencing data that LS neurons highly express mAChR compared to NS cells.

It is reported that layer 2/3 neurons are more selective for the orientation than the deeper cortical layer (Mangini and Pearlman 1980; Niell and Stryker 2008). Several neuron subtypes have showed a visually evoked response profile (Zariwala et al., 2011). In order to differentiate between the response profiles of neuron subtypes, there is a need for other knock-in Cre transgenics such as PV, SST or VIP interneurons, and other cell classes as they emerge through ongoing studies.

## Methods

### Surgical procedures for *in vivo* experiments

Experiments were performed on adult male C57/B16 mice (age 9–11 weeks). Animals were maintained on a 12 h light/12 h dark cycle. Mice were anesthetized using an isoflurane-oxygen mixture (3.0% (*v*/*v*) for induction and 1.5% (*v*/*v*) for maintenance) and kept in a prone position in a stereotaxic apparatus with a heating pad setting to 37–38 °C. After a midline scalp incision, a custom-made plastic chamber was then fixed to the skull with instant adhesive (ALTECO^®^) and dental cement over the left primary visual cortex (V1) according to stereotaxic coordinates (center: ∼2.7 mm lateral and 3.5 mm posterior to the bregma). A small, square craniotomy (2 mm × 2 mm) was made with a pneumatic dental drill. The dura mater was carefully removed by with forceps. Afterwards, the mouse was transferred to and fixed on the recording setup, with light anesthetization with an isoflurane-oxygen mixture of 0.5%–1% (*v*/*v*). The recording chamber was perfused with normal artificial cerebral spinal fluid (ACSF) containing 126 mmol/L NaCl, 3 mmol/L KCl, 1.2 mmol/L NaH_2_PO_4_, 2.4 mmol/L CaCl_2_, 1.3 mmol/L MgCl_2_, 26 mmol/L NaHCO_3_, and 10 mmol/L D-glucose (pH 7.4 when bubbled with 95% oxygen and 5% CO_2_). The temperature of the mouse was kept at ~37 °C throughout the experiment.

### Dye loading

The highly sensitive fluorescent Ca^2+^ indicator Cal-520 AM (Santa Cruz Cat. no. sc-477280) was used for multicellular bolus loading in the V1. Cal-520 AM was dissolved in DMSO with 20% Pluronic F-127 to a final concentration of 250 μmol/L with loading buffer (150 mmol/L NaCl, 2.5 mmol/L KCl, 10 mmol/L HEPES, pH = 7.4) for bolus loading. A micropipette was filled with this solution and inserted coaxially into the cortex and down, reaching a depth of 120 μm below the cortical surface. A pressure pulse [15 min, 0.2 bar (1 bar 100 KPa)] was applied to the pipette to eject the dye-containing solution (Stosiek et al., 2003) The dye of Texas Red 3000 was also dissolved (0.1% solution) in the standard pipette solution and applied through micropipettes similar to those used for injections of AM indicator dyes. We performed the Ca^2+^ imaging ~1 h after dye injection, and the imaging lasted for up to 7 h.

### Visual stimulation

Visual stimuli were presented as described previously (Niell and Stryker, 2008). Briefly, stimuli were displayed with correction on an LCD monitor (7 inch, 1,024 × 768, 60 Hz refresh rate) placed approximately parallel to and 16 cm from the right eye of the mouse, with the visual angle subtended by the monitor (± 23.5° azimuth and ± 26.9° elevation). A paperboard trapezoid cylinder placed between the eye and the screen was used to prevent stray light. The stimuli covered the full screen with black-white drifting square-wave grating presented on a dark grey background. The visual stimuli were generated by a program written in C#. Each trial of visual stimulation sequence started with a stationary six periods of square-wave grating for 1 s. The luminances of white, black and grey were 3.64, 0.11 and 0.12 cd/m^2^, respectively. The grating drifted for 5 s (spatial frequency 0.06 cycles per degree, drifting speed 1.2 cycles per second), with an inter-stimulus interval of 15 s. Each stimulus was repeated 5 times. Neurons that were responsive to the grating less than 4 times were excluded from our data.

### *In vivo* electrophysiology and RNA extraction

To collect an initial dataset of morphology and electrophysiology of neurons, the patch pipette solution contained the following (in mmol/L): 135 K-gluconate, 4 MgCl_2_, 10 phosphocreatine sodium, 10 HEPES, 4 Na_2_-ATP and 0.4 Na_3_-GTP. Approximately 0.1% Texas Red 3000 as a tip indicator was added to the electrode and diffused within the recording neuron during the whole-cell recording, and 0.3% Neurobiotin in electrode for post-recording staining. Patch-clamp pipettes were pulled from borosilicate glass (1.5 mm OD × 0.84 mm ID, VitalSense Scientific Instruments) with electrode resistances ranging from 7 to 10 MΩ. Tight seals (> 1 GΩ) were obtained on cell bodies before rupturing the membrane with negative pressure. Whole cell recordings were conducted with the Axon 700B patch-clamp. The currents were typically digitized at 100 KHz, and macroscopic records were filtered at 2 KHz.

To obtain transcriptome data from single neurons, the internal pipette solution was modified as followed (in mmol/L): 123 K-gluconate, 12 KCl, 10 HEPES, 0.2 EGTA, 4 MgATP, 0.3 NaGTP, 10 sodium phosphocreatine, 20 μg/mL glycogen, and 1 U/μL recombinant RNase inhibitor (Cadwell et al., 2017). Approximately 0.1% Texas Red as a tip indicator and 6 μmol/L calcium indicator dye Oregon Green BAPTA-1 (OGB-1) was added for membrane rupture and inner extraction. All neuron-related reagents were RNase-free. The electrode resistance ranged from 2 to 4 MΩ. The cell membrane was ruptured via negative pressure with a calcium influx indicated by OGB-1. The extracted soma of the neuron was confirmed as the electrode was pulled out of the tissue and then transferred to ice-cold lysis buffer immediately after breaking the tip within the tube. The sample was stored at −80 °C for at most one week before the next step of processing.

### Two-photon imaging

Fluorescence was imaged with a two-photon microscope (Scientific Inc.) equipped with a mode-locked Ti:sapphire laser (MaiTai, Spectra-Physics) and a water immersion objective lens (Apo40×W/NIR, NA 0.8; Nikon). The emitted photons are split into two channels and detected by photomultiplier tubes (PMT), i.e., into a ‘green’ channel for Cal-520 AM and OGB-1 fluorescence (525/50 nm) and a ‘red’ channel for Texas Red fluorescence (620/60 nm). With this configuration of our system, one pixel corresponded to 0.31 × 0.31 μm^2^ in x,y-coordinates. A square region of 160 × 160 μm^2^ (512 × 512 pixels) was scanned at the maximum. The frame rate was 30 frames/s.

### Statistical analysis

The calcium response data were analyzed via ImageJ software (version 1.49g). The cell body pixels were selected manually for each target neuron, as well as the pixels surrounding the cell body as the background. Next, the ratio (∆F/F) of the stimulus-evoked change (∆F) to the average level (F) was calculated as the difference between the average fluorescence signals of the cell body and the background along with the time lapse; the ratio was then normalized with the mean fluorescence across the pixels of each neuron. The calcium spike was defined as an event when the ratio was higher than twice the non-spike level (SNR ≥ 2). The response magnitudes of light-evoked calcium spikes were defined as the mean value of the difference between the peak value during the post-stimulus period (5 s) and the basal average value during the pre-stimulus period (2–4 s), after 5 repetitions.

Electrophysiological data were analyzed with ClampFit software (version 10.2; Axon Instruments) and GraphPad Prism (v.6.0; GraphPad Software). A whole-cell current-voltage curve under a 450-ms ramp command from −90 mV to +60 mV was intended to test the whole-cell current of the neuron. The patterned firing spikes were evoked by a series of 500-ms current pulse injections from −80 pA to 220 pA (40 pA/increment). The field potential was recorded at a current clamp mode with no current injection. Three-dimensional morphological reconstruction was conducted with Imaris software (version 7.6.0, Bitplane). Following the method in previous research (Malagon et al., 2016), EPSC/IPSC, which have fast rising phase and ≥20 pA, was selected manually. These EPSCs/IPSCs were aligned and averaged as the template. We then used template search module in Clampfit (version 10.2) to identify and analyze the EPSCs or IPSCs in original traces. After analyzing, the amplitudes and frequencies data were collected and used for statistical analysis.

### Library construction and sequencing

We converted the RNA collected from the *in vivo* patch-clamped neurons to cDNA for generating the sequencing libraries following the protocol of Smart-seq2 (Picelli et al., 2014). The primers for reverse transcription were referenced from Cadwell’s procedure (Cadwell et al., 2016) After 24 cycles of amplification, approximately 60 ng of purified cDNA was used to construct sequencing libraries using the commercial KAPA HyperPlus Kit protocol (KK8514). The PCR products with different index sequences were pooled together for purification and library construction. Quality control was performed on both the amplified cDNA and the final library using a Fragment Analyser (Thermo Fisher). The DNA was sequenced using an XTEN platform. Investigators were blinded to cell type during library construction and sequencing.

### Read processing and quantification of gene expression

Adapters and low-quality reads were trimmed using Python script AfterQC (Chen et al., 2017). Paired-end reads were aligned to the reference genome GRCm38 primary assembly (Kersey et al., 2018) downloaded from ensemble using STAR (STAR 2.5.3a) (Dobin et al., 2013) with default settings except for the use of setting output type (–outSAMtype) to sort SAM outputs by coordinates. Reads were then counted using featureCounts (featureCounts 1.5.3) (Liao et al., 2014). The R package, scater (single-cell analysis toolkit for gene expression data in R, version 1.6.3) (McCarthy et al., 2017), was employed for quality control, normalization and data visualization. Gene expression was normalized to the number of fragments per kilobase million (FPKM value). Genes that did not have an expression value of at least 1 FPKM across all samples were excluded from further analysis. Moreover, only protein coding genes were considered for subsequent analysis. The filtered dataset contained 20,183 genes in total.

### Quality control

Two cells with cDNA concentration lower than 1,000 pg/μL were discarded. Another two cells with a mitochondrial DNA proportion greater than 25% were omitted. One cell with fewer than 1,000 expressed protein coding genes (which had an expression level lower than 1 FPKM) was excluded as well. The dimensionality of the dataset was then reduced from 63 cells to 58 cells.

### Dimensionality reduction techniques

For dimensionality reduction, principle component analysis (PCA) and t-distributed Stochastic Neighbor Embedding (t-SNE) were performed using function plotPCA from R package scater and function Rtsne (version 0.13), respectively. The dataset was projected onto 2- and 3-dimensional space with both methods.

### Doublet removal

The expression of known marker genes (*Slc17a7*, *Neurod2*, *Gad1*, *Gad2*, *Olig1*, *Olig2*, *Mbp*, *Gfap*, *S100b*, *Aqp4*) was used to name main cell types: excitatory neurons, inhibitory neurons, oligodendrocytes and astrocytes. We firstly checked if any cell from our data had expression of known non-neuron cell type (oligodendrocytes and astrocytes) marker gene through creating co-expression score for oligodendrocytes and astrocytes by combining the following pairs of marker genes:

- $$S_{astro} = Gfap \times S100b \times Aqp4$$
- $$S_{oligo} = Olig1 \times Olig2 \times Mbp$$

Cells with either $S_{astro} > 0 or S_{oligo} > 0$ were omitted (4 cells).

Subsequently, we checked if any cells from our data expressed both excitatory neuron and inhibitory neuron marker genes by computing co-expression score as follows:

- $$S_{ex} = Slc17a7 \times Neurod2$$
- $$S_{in} = Gad1 \times Gad2$$
- $$S_{ex - in} = S_{ex} \times S_{in}$$

Cells with $S_{ex - in} > 0$ were discarded (1 cell), since $S_{ex - in} > 0$ indicated that cells express both excitatory and inhibitory neuron marker genes . In total 5 cells were identified as doublets and removed from the subsequent analysis. Dimensionality reduction analysis with both PCA and t-SNE were then performed on the remained dataset with 53 cells.

### Batch effect analysis

Batch effect analysis was performed under consideration of two factors: (1) cDNA concentration and (2) the dates of experiments. To see if the hierarchical clustering was driven by batch effect produced by the cDNA concentration, we computed the cDNA concentration deviations by subtracting the mean value, followed by assigning dots on the t-SNE plot gradient colors based on their deviations. For dates of experiments, we colored cells based on the experimental dates (15 colors for 15 different experimental dates).

### Heatmap of Pearson correlation matrix

Pearson correlation between light-sensitive and non-light-sensitive subsets was computed. A heatmap was generated using R package pheatmap (version 1.0.8).

### Differential gene expression analysis

Differential gene expression analysis was performed using R package Deseq2 (version 1.18.1) (Love et al., 2014). Genes as P_adj_ < 0.05 and log2FoldChange > 2.5 or log2FoldChange < −2.5 (higher absolute value means higher fold change of corresponding clusters) were identified as differentially expressed genes (DEGs). DEGs were ranked in descending order based on their log2FoldChange value. A heatmap of gene expression from DEGs was generated using R package pheatmap.

### Identification of highly variable genes

The top 200 most expressed genes from the light-sensitive subset were identified using plotQC function from R package scatter by setting type = “highest-expression”. Enriched gene ontology (GO) analysis of those genes was performed using the online tool, DAVID 6.7 (da Huang et al. 2009a, b). Function geom_bar from R package ggplot2 (version 2.2.1) was employed for drawing barplots.

### Ion channel and receptor-related gene analysis

Expression levels of the following ion channel and membrane receptor-related genes across all 53 samples were computed: Na^+^/K^+^-ATPase, voltage-gated Cl^−^, Na^+^, K^+^, Ca^2+^ and Ca^2+^- activated K^+^ (K_Ca_) channel subtypes; ligand-gated ionotropic channels of AMPA-, delta-, kainite- and NMDA-type glutamatergic receptors (Gria, Grid, Grik, and Grin, respectively); GABAergic (GABA), nicotinic acetylcholinergic (nACh) and serotonergic (5-HT) receptors; metabolic Glu (mGlu), GABA (mGABA), 5-HT, muscarinic acetylcholine (mACh), dopamine (DA) and 2-arachidonoylglycerol (2-AG) receptors. Barplots of each gene with the corresponding quantitative expression levels across all samples were generated using R function geom_bar. To determine if the two subsets were significantly different from each other, the *t*-test was employed. Because variances in the two datasets were unequal, the unpaired welch two-sample t-test was performed using R function t.test by setting var.equal = FALSE.

### WGCNA analysis

To find genes highly correlated to external trait, WGCNA analysis was performed using R package ‘WGCNA’ (version 1.63) (Langfelder and Horvath 2008, 2012). Gene co-expression network was constructed based on gene expression correlation. Hierarchical clustering tree (dendogram) of genes was produced using hierarchical clustering method. Densely interconnected branches indicating highly co-expression genes. Modules was identified by cutting the dendrogram using R function cutreeDynamic with deepSplit = 3 and minClusterSize = 10. Modules have similar expression profiles were merged together. Module assignment was followed by quantifying the relationship between modules and the sample trait, where the correlation among them were computed and shown as heatmap. It indicated that the light green color module had the closest association with the sample trait ‘light-sensitive’. Hierarchical clustering of modules and the external trait was computed as well. As shown in the dendrogram and the heatmap, the light green module was significantly correlated to light-sensitive.

### Comparison to high-throughput visual cortex single-cell RNA-seq data

To compare cell types found in our data to previously published visual cortex RNA-seq data which contained over 100,000 cells, we downloaded the gene expression matrix from Hrvatin’s study (2018). The expression matrix was analyzed using the Seurat R package (version 2.2.0) (Butler et al., 2018). Genes had expression in at least two cells and cells expressing a minimum of 200 genes were ketp. The filtered raw expression matrix was than normalized for each cell by the total expression, multiplied by 10^5^, followed by a log-transformation. Variable genes were defined with function FindVariableGenes with default parameters. Variations from difference in total gene number were regressed out with the ScaleData function. Subsequently, PCA and t-SNE were performed for dimensionality reduction with function RunPCA and RunTSNE, respectively. As only neurons from layer 2/3 mouse V1 were collected in our study, we then extracted layer 2/3 cells from the published mouse V1 data based on the expression of *Cux1*, *Cux2* and *Rorb*. Namely, clusters with high expression of *Cux1*, *Cux2* but rare expression of layer 4 marker gene *Rorb* were defined as mouse layer 2/3 clusters. We then performed t-SNE analysis on the these layer 2/3 clusters. After this, we converted our sequencing data from SingleCellExperiment data structure into a Seurat structure. To determine whether each neuron in our data preferentially fall into subclusters in the mouse layer 2/3 V1 cortex, we mapped our data onto the published data based on shared variable genes using function AlignSubspace. We then performed dimensionality reduction and clustering analysis with function RunTSNE and FindClusters on the mapped Seurat object, respectively. Subsequently, we did t-SNE plots by assigning each dot a color based on the major cell types in both datasets (light grey color for the major excitatory neuron cluster and dark grey color for the major interneuron cluster from published data of mouse layer 2/3 V1 cortex, orange color for LS cells, blue color for NS cells). Furthermore, we counted number of cells from our sequencing data founded in each excitatory neuron and interneuron subclusters.

### Data availability

The scRNA-seq data are available in Gene Expression Omnibus under accession number GSE115997.

### Author contributions

Q. W., S. H., and X.W. conceived the project, designed the experiments and wrote the manuscript. J. L. and N.C. P. conducted the animal surgery, calcium imaging and electrophysiology experiments. M. W. and Z. Z. performed single-cell RNA-seq and data analysis. J. L. and L. S. performed 3D reconstruction of neurons. J. Z. maintained the animals. All authors edited and proofed the manuscript.

## Electronic supplementary material

Below is the link to the electronic supplementary material.

Supplementary material 1 (PDF 1836 kb)

Supplementary material 2 (MP4 10173 kb)

Supplementary material 3 (MP4 9331 kb)

Supplementary material 4 (MP4 9835 kb)

Supplementary material 5 (MP4 4083 kb)
