# Supplementary material for: Integrative analysis of in vivo recording with single-cell RNA-seq data reveals molecular properties of light-sensitive neurons in mouse V1
Source: Protein Cell. 2020 Apr 29;11(6):417–32. doi: 10.1007/s13238-020-00720-y (PMC7251024; doi:10.1007/s13238-020-00720-y)
Supplement: Supplementary file 1 — Supplementary material 1 (PDF 1836 kb) [file 13238_2020_720_MOESM1_ESM.pdf]

Figure S1: Confirmation of Cal520 AM labeling in layer 2/3 of V1.

(**A**)Sagittal slice containing the V1 area. Left, Cal520 labeled neurons in V1. Green, Cal-520 staining; blue, DAPI; white dotted line, V1 delimitation. Right, corresponding mouse brain atlas for V1.

(**B**) Confirmation of the recording neuron. Left panel: neurobiotin staining in brain slice. Red for Texas Red staining; blue for DAPI, scale bar: 50 μm. Right panel: 3-D reconstruction of respective neurons. Scale bar, 30 μm.

Figure S2: Basic electrophysiology properties of recorded neurons

(A-B) Statistics of membrane capacitance (A) and input resistance (B) of recorded neurons (n=11 for LS and n=11 for NS). Error bar, mean ± s.e.m. The significance was estimated based on the corrected P value using the unpaired t-test, P >0.05 for non-significance.

Figure S3: Quality control of cDNA libraries.

(**A**) Concentration of all collected samples. Only cells with more than 1,000 pg/μL cDNA were sequenced (cutoff denoted by the dashed line). The excluded samples are indicated in red.

(**B**) Average library sizes generated from the samples screened in (**A**). Dashed line indicates threshold criteria used for sequencing. Poor quality libraries with less than 1,000 genes (FPKM > 1) were excluded (red dots).

(**C**) Mitochondrial proportion of the samples screened in (**B**). The cells with greater than 25% mitochondrial RNA (red dots) were excluded from the sample pool (cutoff denoted by the dashed line).

(D) Co-expression score of cell type oligodendrocytes (upper left), astrocytes (upper right), excitatory neurons and interneurons (bottom), using two-dimensional t-SNE representation.

(**E**) Number of genes detected per neuron using two different expression thresholds.

(**F**) Pairwise Pearson correlation across all detected genes for light- and non-light-sensitive neurons.

(G) Visualization of the cDNA concentration deviations of all cells about their mean by t-SNE. Each dot represents on cell and is colored according to the cDNA concentration deviations (red, high; blue, low).

(H) Visualization of the dates of experiments by t-SNE. Each dot represents a cell and is colored based on the date, when they were collected.

Figure S4: Marker gene expression overlaid onto t-SNE map.

(**A**) Marker gene expression of cortical neurons, including pyramidal cells and interneurons, using two-dimensional t-SNE representation.

(**B**) Cortical layer 2/3 marker gene expression overlaid onto the t-SNE map.

(**C**) Reported enriched V1 gene expression overlaid onto the t-SNE map.

Figure S5: Comparison to high-throughput visual cortex single-cell RNA-seq data.

(A) Visualization of excitatory neurons from published visual cortex scRNA-seq data via t-SNE. Each dot represents an individual cell. Cells were annotated to 30 subclusters.

(B) Visualization of the expression of known marker genes for excitatory neurons (*Slc17a7*), layer 2/3 (*Cux1* and *Cux2*) and layer 4 (*Rorb*) via t-SNE. Cells were colored according to the gene expression levels (red, high; grey, low).

(C) Visualization of interneurons from published visual cortex scRNA-seq data via t-SNE. Each dot represents an individual cell. Cells were annotated to 14 subclusters.

(D) Visualization of the expression of known marker gene for interneuron (*Gad1*), layer 2/3 (*Cux1* and

Cux2) and layer 4 (*Rorb*) via t-SNE. Cells were colored according to the gene expression levels (red, high; grey, low).

(E) Integration of our data with layer 2/3 excitatory neurons and interneurons from published data was visualized via t-SNE. Excitatory neurons and interneurons were annotated into 10 and 4 subclusters, respectively.

(F) The distributions of cells from our study were visualized by t-SNE. Dots were colored based on the major cell type annotated in our sequencing dataset and the published dataset, (excitatory neurons in the published data, light grey; interneurons in the published data, grey; LS neurons from our data, orange; NS neurons from our data, blue).

(G) Bar plot showing the occupancy of LS and NS neurons in the major excitatory neuron and interneuron cluster.

(H) The constitutions of the excitatory neuron and interneuron subclusters were shown in pie chart. The number of cells from our sequencing data founded in each subcluster was labeled.

Figure S6: Rtn4r do not block neuron activity in general.

(A-B) Statistical histogram of the calcium response counts in normal neurons (light response ≤ 2) under the NEP1-40 application, n=15 neurons from 4 mice (A) and vehicle as a control, n=16 neurons from 3 mice (B). Error bar, mean ± s.e.m. The significance was estimated based on the corrected P value using the unpaired t-test, P >0.05 for non-significance.

Video S1: Two-photon in vivo calcium imaging of V1 neurons in light-evoked mouse, followed by LS neuron soma extraction.

Cal 520-labeled cells in layer 2/3 of mouse V1 were recorded to measure the calcium response to the 5-time repeated light stimulus, under the 'green' channel of the two-photon microscope. A light-responding neuron was subsequently targeted to extract the contents of the soma via a glass cuspidal electrode. The light response cells are indicated by yellow arrows, and the target cell is indicated by the red arrow. The frame of calcium imaging was shifted to the left for a better view of the soma extraction. The cells marked with white arrowheads are coordinate references for locating the target cell. Scale bar, 20 µm.

Video S2: Two-photon in vivo whole-cell configuration.

Left panel: 5-minute movie of whole-cell patch clamp configuration with the cuspidal electrode under the 'red' channel of the two-photon microscope. The shadow of the target cell is visible. Scale bar, 20 µm. Right panel: the corresponding membrane test monitor from the recording software of Clampex. The cells marked with white arrowheads are coordinate references for the target cell. Scale bar, 20 µm.

Video S3: In vivo dual recording of light-evoked calcium response and action potential firing.

Left panel: the video shows the calcium response to light stimulation of a whole-cell patched neuron (Cal-520 labeled), under the 'green' channel of the two-photon microscope. The target neuron is indicted by a yellow arrow, and the position of the glass electrode is marked. Scale bar, 20 µm. Right panel: the calcium response and the action potential firing are dynamically represented in the 'calcium signal' and 'electrophysiological signal' windows, respectively.

Video S4: 3-D morphological reconstruction of a neuron in layer 2/3 of V1.

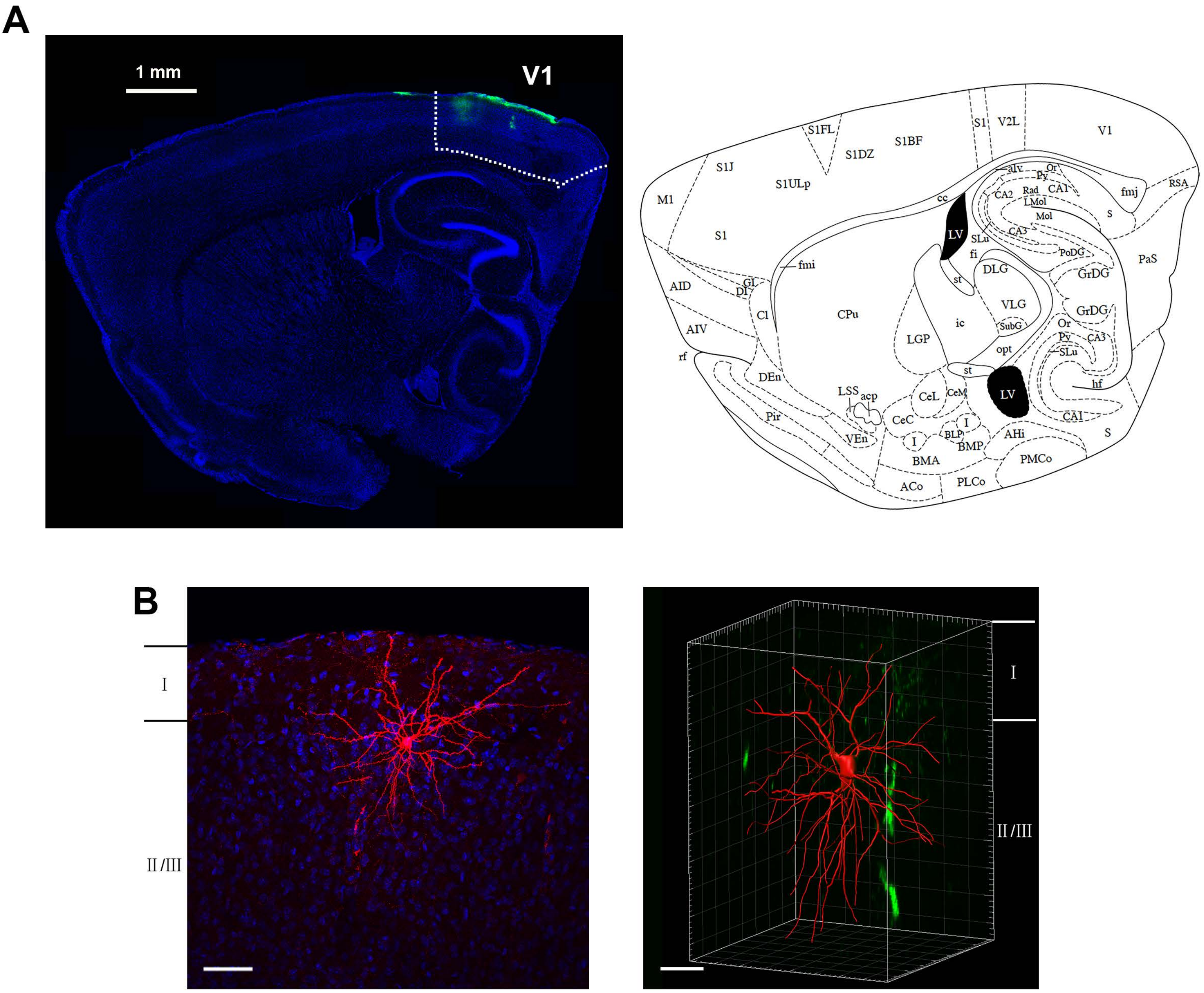

Supplementary Figure 1

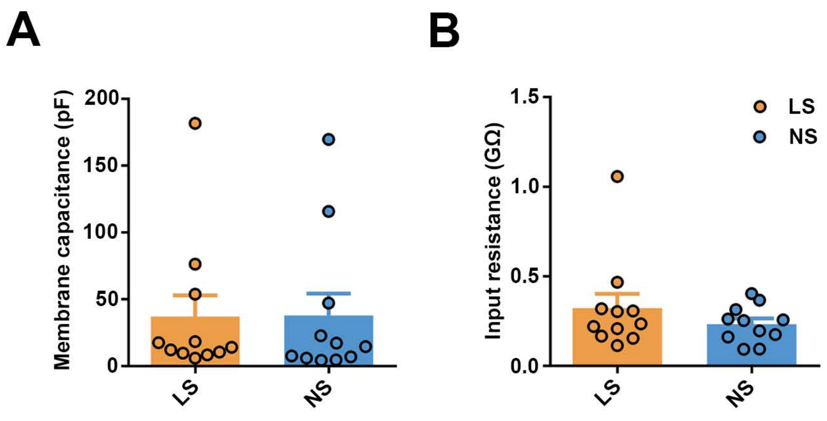

Supplementary Figure 2

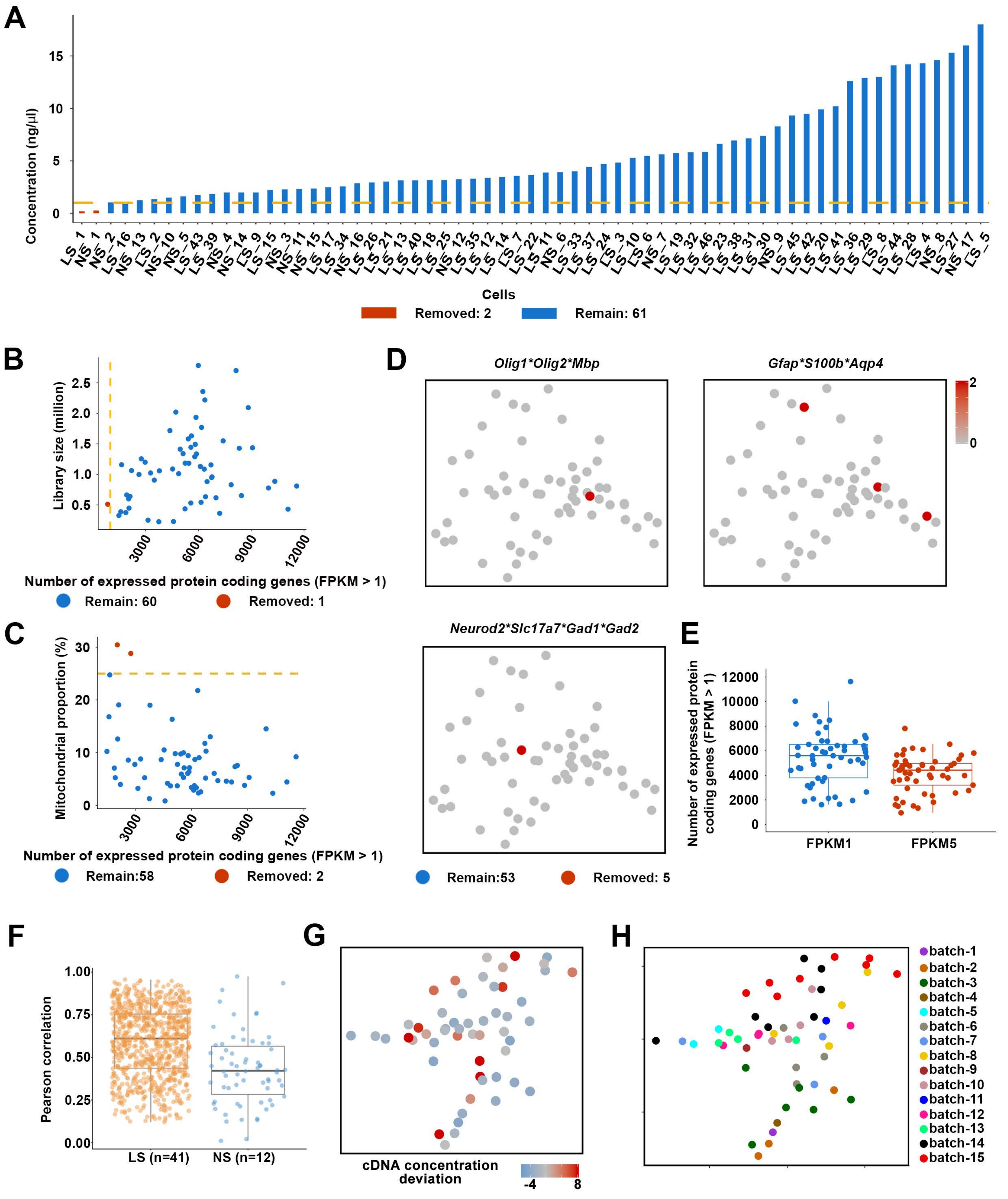

Supplementary Figure 3

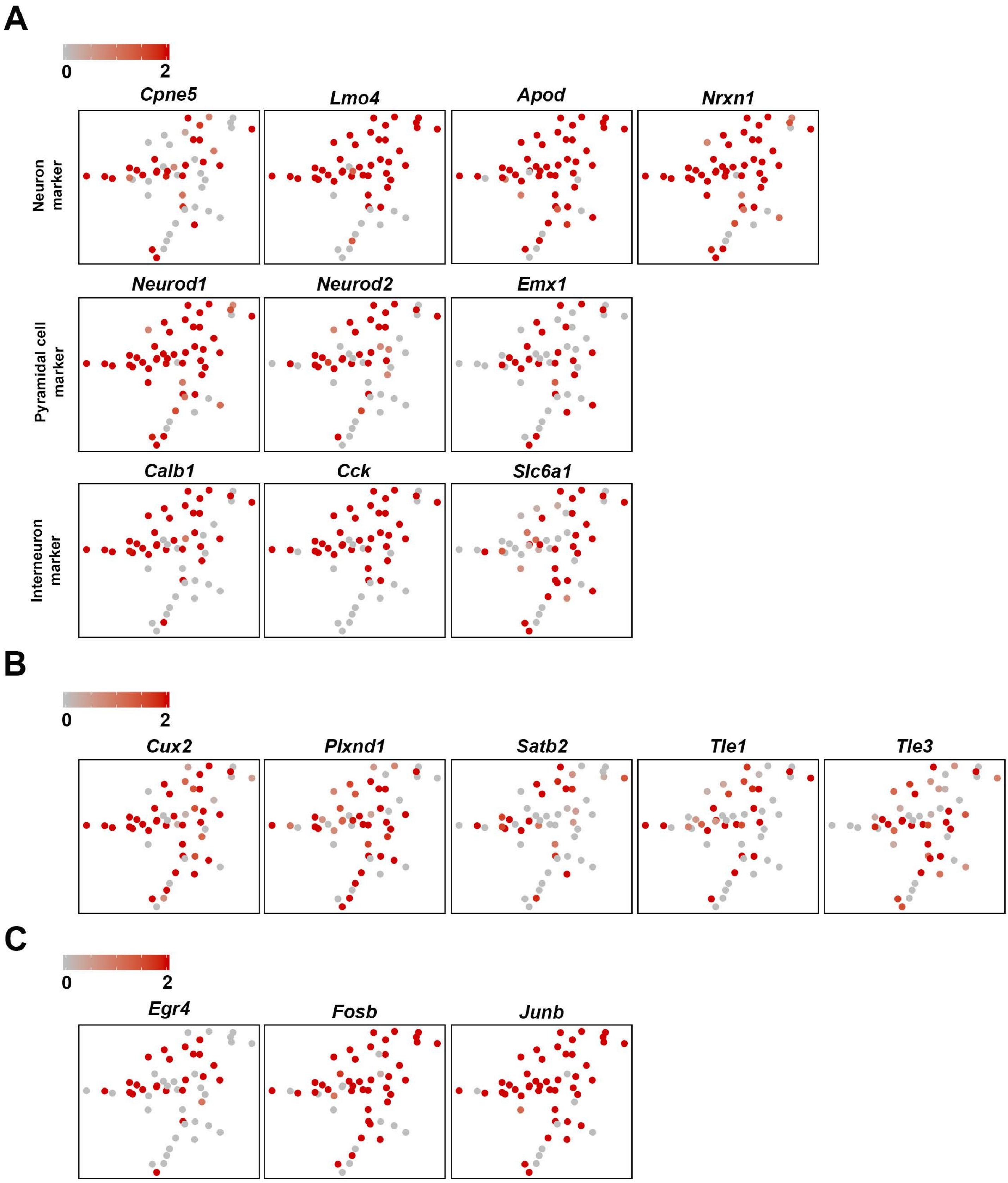

Supplementary Figure 4

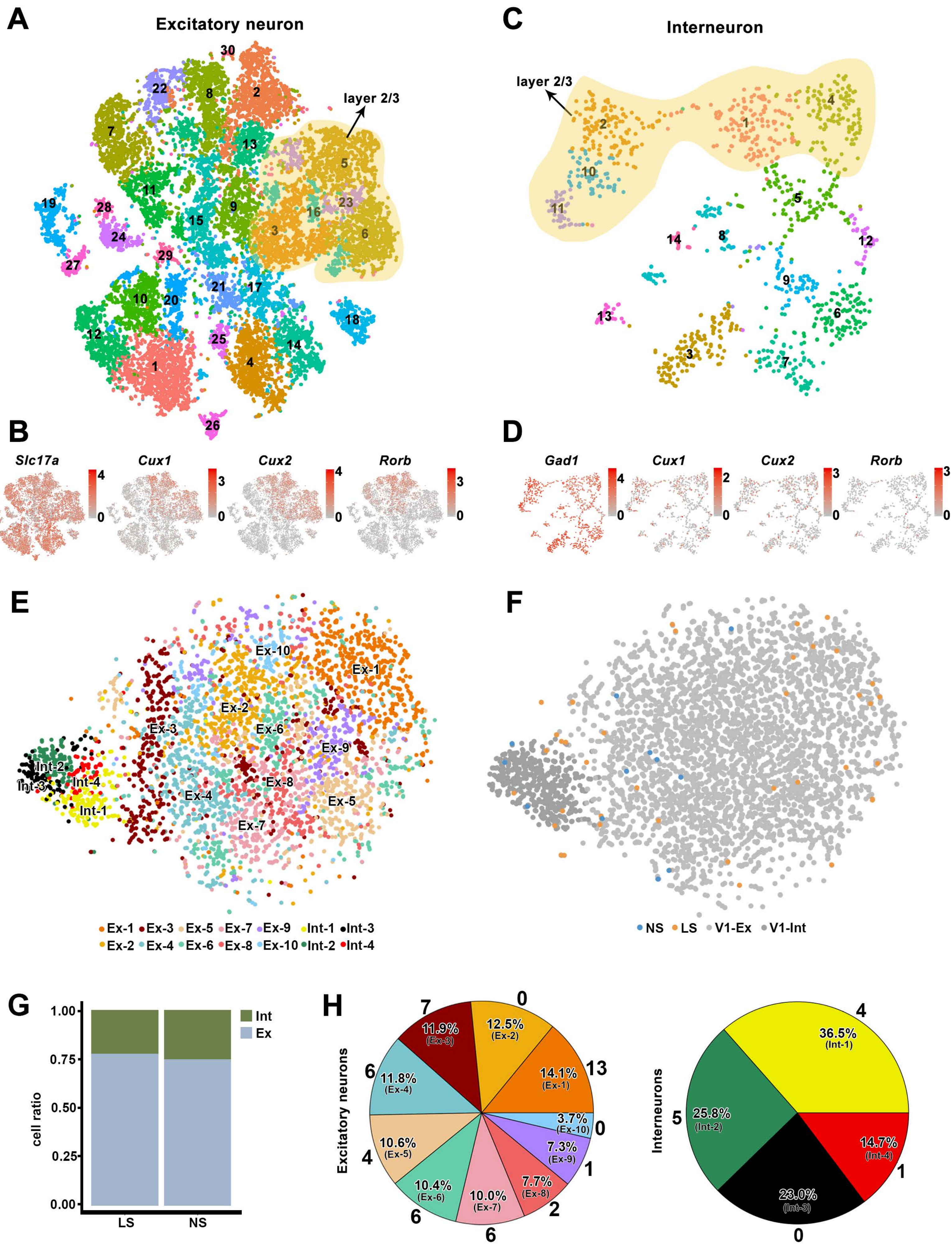

Supplementary Figure 5

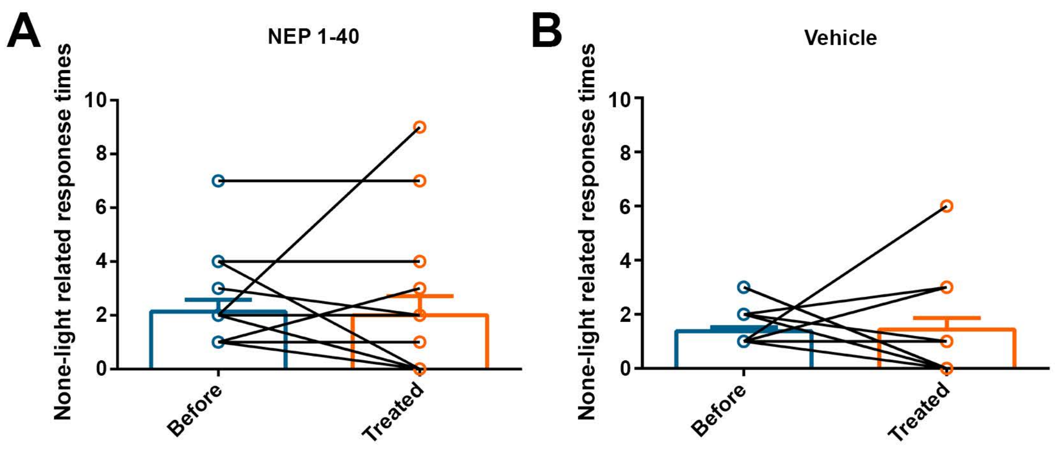

Supplementary Figure 6
